# Becoming a doctor: using social constructivism and situated learning to understand the clinical clerkship experiences of undergraduate medical students

**DOI:** 10.1186/s12909-024-05113-x

**Published:** 2024-03-05

**Authors:** Hyena Cho, Hyeyoon Jeong, Jihye Yu, Janghoon Lee, Hyun Joo Jung

**Affiliations:** 1 Department of Medical Education, Ajou University School of Medicine, Suwon, South Korea; 2 Department of Pediatrics, Ajou University School of Medicine, Suwon, South Korea

**Keywords:** Situated learning theory, Social theory in learning, Clinical clerkship, Legitimated peripheral participation, Negotiation of meaning, Basic qualitative study

## Abstract

**Background:**

Despite the emphasis on the uniqueness and educational importance of clinical clerkships in medical education, there is a lack of deep understanding of their educational process and outcomes. Especially due to an inherent trait of clinical clerkships which requires participation in the workplace outside the classroom, it is difficult to fully comprehend their educational potential using traditional learning perspectives such as imbibing outside knowledge. Accordingly, this study aims to explore the experiences of a rotation-based clerkship of medical school students from the perspective of social constructivism of learning, which can empirically examine what and how medical students learn during clinical clerkship in South Korea. By providing an insight into the workings of the clerkship process, this study contributes to a better understanding of how a learning-friendly environment can be cultivated at clinical clerkships.

**Methods:**

The study utilized a basic qualitative study to understand what and how medical students learn during their clinical clerkships. Semi-structured, in-depth individual interviews were conducted with eight sixth-graders who had experienced a two-year clerkship at Ajou University Medical School. Data were analyzed based on Lave and Wenger’s situated learning theory and Wenger’s social theory in learning.

**Results:**

We found that the medical students had developed different aspects of their professional identities such as values, functionality, career decisions, sociality, and situating during their clinical clerkships. Further, professional identity was formed through a combination of participation and reification—the processes involved in the negotiation of meaning. This combination was facilitated by the students’ first experience and relationships with professors, classmates, and patients. Finally, non-learning occurred in the context of over-participation (learning anxiety and alienation) or over-reification (evaluation and e-portfolio).

**Conclusions:**

This study revealed five sub-professional identities and their formation process from the learners’ perspective, thereby uncovering the unique learning characteristics and advantages of rotated-based clerkship and contributing to a further understanding of how gradual improvements can be made to the traditional clerkship education of medical students.

## Background

Clinical clerkship is an educational process where medical students learn and gain experience in healthcare settings under the supervision of a skilled medical practitioner. The knowledge, skills, and attitudes acquired during clerkship learning represent a unique educational methodology that cannot be replaced by any other methods [1]. Arthur Dean Bevans, the first chair of the American Medical Association Council, underscored the educational significance of clinical clerkship in medical education by stating, “Competent practitioners of medicine can be trained in only one way, and that is in the hospital and the dispensary” [2]. Greater recognition of the importance of clerkship has led to an increase in medical education research on clerkship practice. In particular, the longitudinal integrated clerkship (LIC) style of learning is being highlighted as a promising direction for clinical clerkship to the extent of being considered “the most significant educational innovation in clinical education to have occurred in our lifetimes” [3] and as an alternative approach to the traditional rotation-based clerkship (RBC), garnering extensive research. LIC programs initially emerged in rural medical schools and those comprising small student populations [4–5]. There, these programs aimed to improve students’ learning engagement and the quality of their relationships with their patients and professors based on the principle of continuity of care [6]. In recent years, there has been a spurt of research activity to demonstrate the superiority of LIC, especially regarding academic achievement, compared to RBC [7–9].

Consequently, studies on clerkship practice have highlighted multiple concerns with RBC, including difficulty learning patient-centered care, communication skills, and professionalism [6]; however, some maintain that there is no difference in academic achievement between RBC and LIC [8, 10]. Nevertheless, RBC is routinely distinguished from LIC and discussed superficially in terms of its educational outcomes such as academic achievement and learner satisfaction. Criticisms of RBC might arise from delineating the boundaries of cognitivism within the framework of traditional educational discourses based on cognitive learning theories. In particular, considering that clerkship intrinsically aligns with the prototype of situational and context-specific apprenticeship, traditional learning approaches—oriented toward the acquisition of knowledge framed as a cognitive and emotional phenomenon experienced by learners [11]—can obstruct a thorough comprehension of the full breadth of the practical learning process. Further complicating matters, misunderstandings have emerged due to the lack of in-depth analysis in prior research on the implementation of the RBC process. It has also been referred to as a “black box” because it remains unclear what clinical clerkships actually entail [12–14]. Hence, additional research is warranted. Therefore, this study ventured beyond traditional learning discourses, aiming for an in-depth exploration of the essence of RBC from a social constructivist perspective on learning. Specifically, it was aimed to empirically examine what and how medical students learn in RBC, drawing from their educational experiences. By clarifying the workings of clinical clerkship, this study contributes to a better understanding of how a learning-friendly environment can be created at clinical clerkships. The research questions are as follows: [RQ1] What are the learning outcomes of RBC? [RQ2] What environments promote these learning outcomes? And [RQ3] What environments hinder learning in RBC?

These research questions were explored based on analytical frameworks from representative theories of social constructivism that explain and understand learning. Social constructivism theory is a model of interactive learning within cultural and social contexts [15] and has the advantage of viewing learning from a holistic perspective, extending beyond the individual cognitive domain to include emotional and social dimensions [16]. Specifically, Lave and Wenger’s concept of “legitimate peripheral participation” [17] from their situated learning theory, originating from investigations of apprenticeship intrinsic to clerkship learning, was utilized. Additionally, the “negotiation of meaning” [18] from Wenger’s social theory of learning, an evolution of the situated learning theory, was incorporated. Situated learning theory offers a comprehensive and process-oriented perspective that covers the cognitive, emotional, and social facets of learning [16], based on the premise that all learning is “situated” [17]. A pivotal element of the theory, legitimate peripheral participation, describes learning as a process by which novices in apprenticeship transition from peripheral participants in communities of practice (CoP) to full participants occupying the core position of the community. This theory suggests that learning is not merely about acquiring knowledge and skills of the world but is more about the way of being in the world. Consequently, even if participants in a given context have no explicit intent to teach or learn a specific skill, the structure of their social participation can inherently promote an activity recognized as learning [19]. Lave and Wenger elucidated the conventional relationship between a master and apprentice (or teacher and student), characterizing it in terms of shifts in both practice (external actions) and identity (internal perceptions).

Wenger further developed the situated learning theory, refining it into a social theory of learning. In this process, he redefined learning both as a means of generating practice and as a means for newcomers to develop their identity [18]. The term “practice” denotes the interactive process among members of communities and marks the beginning of the phenomenon where knowledge is shaped and the ensuing change in identity formation [20]. The term “identity” refers to the outcome of learning that is molded through practice [20]. It is a process-oriented domain, continuously evolving, and shapes the understanding of “who I am” based on the learner’s self-situating in the world [20]. Meanwhile, practice is concretized by the concept of the “negotiation of meaning” by which meaning is endowed to our experience gained through our active participation in the world [21]. The negotiation process is dynamic and on-the-spot situational, occurring as a blend of participation and reification. “Participation” means actions and interactions, while “reification” refers to the artifacts (tools, terms, symbols, rules, documents, concepts, theories, etc.) formed as the negotiation of meaning is organized [20]. In the negotiation of meaning, these two are in a complementary relationship [20–21].

In summary, this study aimed to conduct an in-depth empirical exploration of RBC, focusing on its inherent nature as context-specific and situational learning, from the perspective of social constructivism. This study attempted to gain a comprehensive understanding of clerkship learning, which has hitherto been understood superficially and fragmentarily. By deeply interpreting the processual domain of learning in RBC from the perspective of the learners, the true subjects of learning, this study aimed to shed new light on RBC and suggest improvements in the quality of education from a fresh viewpoint.

## Methods

### Study design

This study used a basic qualitative study design to analyze in depth the experiences of medical students and examine processes during their RBC. Basic qualitative study enables an in-depth exploration of people’s lived experiences and meaning-making [22], and is appropriate to employ when solving a problem of practice [23]. The purpose of this research design is particularly well-fitted to attain an in-depth understanding of effective educational processes [24]. By describing medical students’ experiences during their clinical clerkship, it was aimed to comprehend and interpret how they shape their world and what meanings they ascribe to their experiences from their own perspective [22].

### Participants

This study enrolled eight sixth-year students from Ajou University School of Medicine in the 2022 academic year who had completed a two-year RBC program. While the sample size is not generally predetermined in qualitative research [25], most importantly, the sample size is sufficient when additional interviews do not result in the identification of any new concepts [25]. Given this, all researchers of the current study agreed that data saturation had been achieved after the eighth interview.

Purposive sampling [26] was employed to ensure participants had firsthand experiences pertinent to this study and the ability to articulate them effectively. The inclusion criteria were: (1) Mandatorily, sixth-year medical students who had finished both the mandatory RBC (covering internal medicine, surgery, obstetrics and gynecology, pediatrics, mental health, family medicine, and neurology practices) and the elective clerkship. By including experiences from both the mandatory and elective clinical clerkships over two years, it was intended to delve into the social significance of experiences within varied groups during elective clinical clerkships [27] and to factor in diversity within the qualitative research sample [28]. (2) Preferably, individuals who could actively cooperate as study participants and provide profound insights were prioritized. While voluntary participation was encouraged, recommendations from the clerkship coordinator were also considered. Only those willing to speak openly about their RBC experiences were considered. Consequently, eight participants were finalized. This study was approved by the Institutional Review Board (IRB) of Ajou University Hospital (Ethics Consent No. AJOUIRB-SB-2022-462).

### Data collection

This study primarily used data collected through in-depth interviews with participants. Additionally, researcher-generated notes, reflective journals, and the guidebook from Ajou University School of Medicine served as supplementary materials. The in-depth interviews were conducted face-to-face over approximately three months, from October 17 to December 13, 2022, with each participant interviewed once. On average, individual interviews lasted about 90 min and were based on semi-structured questionnaires. For instance, based on the interviewees’ responses to the common question “Were there any notable episodes during your clinical clerkship?” various follow-up questions were asked regarding noteworthy individuals or tools, interpersonal relationships, factors facilitating or hindering learning, and emotions, thoughts, and reflections from the episodes experienced. The interviews were conducted in a natural and relaxed atmosphere in locations such as lecture halls and lounges at the clerkship education center, ensuring no presence of other students, professors, or any stakeholders related to the clerkship. Prior to an in-depth interview session, the researcher explained the study topic, its objectives, and the research method, including recording and transcription. Then the participants completed the IRB-approved informed consent form. Immediately after the interview, transcripts and reflective journals were composed to capture the mood during the interview session and any non-verbal implications from the interviewees. After the initial interview, any additional queries to the interviewees were addressed periodically using electronic methods such as text messages and emails.

### Analysis

This study used thematic analysis and Lochmiller and Lester’s seven phases to be employed when conducting a systematic qualitative analysis [29]. Thematic analysis is conceived as “an umbrella term, designating sometimes quite different approaches aimed at identifying patterns (‘themes’) across qualitative data sets” [30]. The theoretical flexibility of thematic analysis means that it can generate either a theory-driven or a data-driven set of findings and hence be utilized to address diverse research questions [31]. The seven phases of thematic analysis generally include preparing and organizing the data, transcribing the data, becoming familiar with the data corpus, memoing the data, coding the data, producing categories and themes from underlying coded passages, and making the analysis process transparent [32]. To enhance the validity and reliability of the analysis, the research findings were then subjected to expert consultations in addition to the researcher’s continuous reflective introspection and cross-referencing of multiple data sources. Continuous interpretive exchanges also took place among the co-researchers, and a results review process was carried out with the participants concerned, focusing on enhancing reliability and validity. This series of processes occurred in a cyclical rather than in a fragmented and linear manner [32].

Language should be carefully considered in qualitative research to conserve underlying meanings [33]. Therefore, the process of translating the interview transcripts from Korean to English was carefully conducted with the assistance of a professional translation company (Editage). A translation team of four bilingual individuals (i.e., English and Korean) was formed for the translation and back-translation processes. The researchers who had conducted the in-depth interviews and analyzed data for this study operated as translation moderators in cooperation with professional translators [33].

## Results

For medical students, clinical clerkships represented the journey of transitioning into a doctor in the world of medicine. Operating under the premise of legitimate peripheral participation across various healthcare providers, students molded their identity through diverse relationship-building approaches. Table 1 presents the study’s results derived from the research questions.

**Table 1:** Themes and overview of the categories and sub-categories

Themes	Categories	Sub-categories
Destination for Learning: Learning Outcomes	Identity of becoming a doctor	Wearing a white coat
		Values
		Functionality
		Career decision
	Widening an identity to others and society	Sociality
		Situating
Learning Journey: Facilitators and Barriers	Negotiation of meaning	Learning process as participation
		Learning process as reification
	Promoting the negotiation	From first experience to learning
		From relationship to learning
	Inhibiting the negotiation	Over-participation as non-learning
		Over-reification as non-learning

### Destination for learning: learning outcomes

#### Identity of becoming a doctor

The initiation of clerkship learning, symbolized by “wearing a white coat,” holds profound personal significance for medical students at the individual level. At the organizational level, this act confers legitimacy upon students as trainees, allowing for peripheral participation within healthcare settings. Essentially, the white coat stands as a badge of identity and membership within the healthcare community for medical students. Prior to their clinical clerkship, students harbor aspirations, expectations, and a sense of responsibility toward their impending role as doctors. Moreover, the white coat ceremony offers them an inaugural opportunity to participate and learn within a real hospital organization.

To be honest, wearing a white coat and walking around the hospital felt like the realization of a dream I’ve had ever since admission. It was also filled with anticipation (Participant 5).

When I see my seniors in their white coats, they look impressive. In the hospital, our work uniforms are distinctively green. Only the trainee students wear green, only the medical students… Just seeing our seniors in white evokes a sense of envy (Participant 6).

Through RBC, students continuously adjusted and changed the identity of the white coat and learned the values, functionality, and career decisions of becoming a doctor on a personal level. Values were solidified by the ethics and responsibility of medical service and generated meanings like “helping others.” Going a step further, they materialized their unique ideals of a physician, such as “a doctor with good judgment,” “a trendsetting doctor,” and “a charismatic doctor” as role models.

It’s hard to clearly say that it’s a particular lesson. But there, I learned a lot about the attitude, responsibility, ethics, and demeanor of a healthcare professional. I’ve felt the real issues and patients’ financial situations and felt a kind of societal responsibility (Participant 1).

These days, there’s an emphasis on attentive doctors. I think those things are necessary, and we need them for further progress. But basically, skills should be the foundation. You need to be armed with knowledge and have basic techniques. Once you have that foundation, then you can add other qualities such as having attentive and empathetic attitudes toward patients. I want to be a doctor who excels in skills (Participant 3).

Functionality refers to the ability to operate effectively in various situations one encounters through a thorough understanding of real-world problems and the capacity to address genuine challenges [18]. Drawing from their educational experiences prior to the clinical clerkship, students had the opportunity to recontextualize their medical knowledge, comprehension, and skills within actual healthcare scenarios as trainees. Furthermore, the clerkship equipped them with specialized know-how for unique situations. Students could develop clinical competence, decision-making, and practical skills.

In my first year of the regular course, when I was first introduced to medicine, I found it difficult to differentiate what was crucial from what was less so. As we prepared for the state exam in our sixth year, our main resources were textbooks. However, these textbooks often presented an inflated amount of information, much of which wasn’t necessary for the state exam. For the first two years, I grappled with understanding what was truly essential and determining the best way to study. It was only in the 5th and 6th years when our focus shifted primarily towards preparing for the state exam. Then I began to grasp the concepts our professor had emphasized in the earlier years and to discern what I needed to learn for the exam and how to tackle it (Participant 3).

It was when I was doing my rounds in the emergency room. A patient presented with difficulty breathing, feeling suffocated, and complaining of chest pain. The patient was suspected of having a pulmonary embolism, a condition where a blood clot obstructs the flow of blood to the lungs, causing an issue with oxygen exchange. Despite this suspicion, the patient firmly stated, “I won’t get a CT scan.” I wondered how I would handle the situation if I were a specialist. I pondered deeply on the appropriate response to such situations (Participant 7).

Furthermore, they actively designed their own career paths, ranging from short-term decisions regarding elective subjects and internships to long-term planning for their future roles. Career decisions were strongly influenced by RBC, resulting in the convergence of career choices prompted by their positive or negative emotional experiences during each departmental clerkship. This process of finding departments that aligned with their aptitude and interests, and narrowing down their options by excluding unsuitable ones through clinical clerkship, echoes the findings of a previous study [34]. Additionally, the emergence of expanding career paths due to the discovery of a new self was noted.

In the case of psychiatry, even up to my second year of the main course, there were departments I was interested in. This is because I had not found it that interesting until the second year of the regular course. This changed during the clinical clerkship in the third year. I found the department itself so fascinating that I developed a new interest (Participant 4).

Surgery can end within an hour, but longer ones can take up to 3 or 4 h. During such times, standing continuously and maintaining my focus allowed me to evaluate my concentration levels and assess if I’m suited for surgical departments. I believe it was a valuable time for making such decisions (Participant 7).

As time goes on, I’ll be narrowing down my department choices and deciding what I want to do in the future. However, as a student, it’s a period where I can keep all possibilities open and hope for anything. That’s why I wanted to see as much as I could (Participant 1).

#### Widening an identity to others and society

The identity formed at a personal level continuously evolved and expanded through interactions with the external environment, establishing connections with others and society and extending toward “sociality” and “situating” within the healthcare organization. Particularly, the development of “sociality,” defined as the capacity to facilitate and aptly function in various forms of social interactions among individuals [16], stood out as a significant educational transformation that learners themselves could recognize. Through RBC, encounters with healthcare professionals and patients in a real-life healthcare workplace, coupled with insights from group activities with peers, aided students in seamlessly integrating into a broader social context and community. Students had the opportunity to develop their emotional intelligence to form sociality.

I believe that stepping out to work as a doctor and interacting with others taught me a great deal about how to deal with people. This aspect of handling people is what stands out the most to me. We get the opportunity to observe various professors across different departments. Observing how different individuals interact with others was one of the most valuable parts of the clerkship for me (Participant 6).

The most significant learning for me was definitely interpersonal relationships. It was the first time I interacted with professors and others beyond just being a student attending lectures and mingling with my peers. I often caught myself worrying about such things (Participant 8).

Newcomers start from a peripheral position within a group, striving to understand the overall context of the community they are about to join, and experiencing the process of situating themselves within that context [18]. This experience contributed to students’ adaptation to the organization by helping them grasp the roles of individuals within the healthcare organization, understand their own roles in relation to these individuals, and learn about the structures and operations of the healthcare organization formed by these interactions.

From a practical standpoint, it was good to see how a hospital functions as an organization. Even if I don’t become a professor, I’ll still be affiliated with a hospital for the next few years. So, the experience was meaningful. Grasping how a hospital operates, or more broadly, understanding the workings of a healthcare organization, holds significant value to me (Participant 2).

At first, I was quite worried. I wasn’t sure how to act in the hospital, or what kind of attitude or demeanor I should show. I didn’t even know what could get me in trouble, so that was stressful. But as time went on, I felt less worried. It seems I learned through experience (Participant 5).

### Learning journey: facilitators and barriers

#### Negotiation of meaning

Learning emerged from the negotiation of meaning, anchored in the mutually complementary relationship between participation and reification. First, there was a “learning process as participation” that took various forms, from passive learning activities such as observing outpatient consultations and surgeries and attending lectures and conferences, to active learning activities such as lab testing practice, initial diagnoses, and presentations. When learning leaned more toward passive activities, there was a more pronounced development of values and attitudes. Conversely, when learning leaned more toward active activities, there was a more noticeable negotiation of meaning, characteristic of cognitive development. In passive learning, the focus was on observing the professor, with the educational significance stemming from role modeling. In active learning, learners, when situated in specific contexts, specialized contextual knowledge through their direct actions.

As always, the professors’ bearing in the operating room is astonishing. For me, it was an insurmountable distance, and I felt I would never be like that. They were so dexterous in handling the anatomical structures, cleanly excising, and suturing. I felt I might not be able to do that. I find it admirable, and at times, it evokes a sense of awe in me (Participant 5).

As for the knowledge aspect, it’s like revisiting what I learned. So, what I saw during the clerkship especially lingers in my memory. Yet, what I presented during the clinical clerkship stays more vividly in my mind. What I saw or learned has more lasting effects than what I heard. For example, surgeries. These experiences are what I remember the most (Participant 8).

The “learning process as reification” was also observed. From prep notes written directly by the students, which are informal records, to official creations such as observation records, initial diagnosis records, and presentation materials, as well as the use of online media such as e-portfolios and online cafes, various types of reification assisted in the negotiation of meaning. Given that reification requires significant intellectual effort from the learner, difficulties were observed in the early stages of learning. On the other hand, reified artifacts were easily shared, and the online platform, which expands both time and space, provided a learning environment more conducive to the negotiation of meaning.

Above all, the presentations were a novel experience. We were tasked with giving compact presentations for nearly a year. There were times when I’d have to deliver three to four presentations in just one week, which was quite demanding. Preparing a presentation in itself is arduous. Initially, there’s a specific format we need to adhere to, especially in medical school where we have both case and topic presentations. For topic presentations, there’s a predetermined structure that we must follow. On top of that, it’s crucial to highlight any unique aspects and incorporate medical terminologies to ensure the patient’s condition is depicted precisely. At the outset, expressing these was rather challenging (Participant 6).

We’re consistently tasked with assignments. For instance, upon exiting the operating room, we’re required to write surgery observation records; after conducting an initial diagnosis, we need to complete a corresponding record. So, we usually finish these tasks in the evening. What is more, our year group was provided an online system called e-portfolio, which mandates the inclusion of daily, weekly, and even final reflections (Participant 1).

One feature I particularly appreciated was our online cafe. This platform housed various materials and presentations, allowing us access to works both from our peers and seniors. So, during my early days, I frequently turned to these resources to get ideas for my own presentations (Participant 4).

#### Promoting the negotiation

Learners who have experienced something tend to reflect on the meaning that experience holds for them [18]. In the clinical clerkship experience, the catalysts for reflections leading to active negotiation of meaning were confirmed to be the experiential characteristics of “from first experience to learning” and “from relationship to learning,” which influenced the students. Initially, students who were introduced to a healthcare organization utilized the sequential workplace changes in the RBC as a nourishment to foster learning. The first experience triggered a strong learning emotion in the learners, enhancing the sustainability of learning and the possibility of transfer. A unique characteristic of RBC, where every moment is a first experience, continuously placed the learners at the periphery, providing an environment that allowed them to experience the constant changes in the CoP. This continuous peripheral participation induced a state of learning tension among the students, maintaining a high motivational state for the negotiation of meaning.

I believe that our first experiences are always the most memorable. In this context, the seamless string of first experiences in clerkship stands out as its advantage. I distinctly recall the first cesarean section I witnessed in the obstetrics and gynecology department, especially the moment the baby was delivered. Whenever I carry out a procedure or technique on a patient, that initial experience tends to linger in my memory (Participant 2).

Actually, it was more fun than it was a challenge. Clerkship is indeed enjoyable. Even though the department changes every week, which can be quite chaotic, it also means that there’s always something new, making it very engaging. I remember feeling incredibly excited the first time I entered the operating room (Participant 1).

Human beings form relationships, and forming relationships is a human condition [35]. As individuals who teach and learn, forming relationships constantly shapes and constructs the evolving self-identity [35]. During relationship-building with professors, peers, and patients encountered in the educational environment, students found themselves in unique situations that promoted the negotiation of meaning within those relationships. In the classic learning relationship with professors, learners appeared to react sensitively to the professors’ responses to their actions. The praise and support from professors satisfied the learner’s need for acknowledgment, cultivated a trust-based atmosphere, and continuously facilitated meaning formation. Notably, it was observed that by merely showing authenticity in participation in the community of practice, professors could enhance learners’ meaning formation.

When presenting to the professor and suggesting, “I believe this patient should be treated this way,” and the professor responds with, “That seems like a good approach,” it gives me a sense of acknowledgment. It assures me that had I treated an actual patient in that manner, it would have been effective. The mere alignment of my thoughts with the professor gives me the feeling that I’ve done really well (Participant 4).

What stands out most in my memory is that the professors didn’t put undue pressure on us students, though they posed many questions. Even if I couldn’t provide an answer, they never reprimanded me or asked, “Haven’t you studied this?” The method of retracing the logic or reasoning behind things aided in my retention of the content. It allowed me to identify areas I was unfamiliar with, my limitations, and the scope of my understanding, prompting me to revisit and study the material again at home (Participant 7).

We naturally look up to professors for their knowledge and skills accumulated over a long period of study and experience. They are all so professional and extraordinary, and try to teach students so many things. This made me admire them more and want to learn more from them (Participant 3).

For learners, peers played a dual role in enhancing learning. Peers were observed to not only monitor students’ reputations but also to provide a sense of belonging and solidarity through their support and encouragement. This ensured the maintenance and enhancement of the negotiation of meaning. In alignment with previous studies, which highlighted the positive impact of mutual peer support on students’ motivation, emotions, and academic achievements, this study also unveiled a mutually beneficial relationship in which peers take on the role of observers in the process of identity formation [36–39].

In the early stages of clerkship, there was a strong sense of, “I will do my utmost.” I didn’t want to show my inadequacies in the new environment to my peers or to myself. Despite the challenges, working hard made me feel content and happy in my own way (Participant 2).

When I saw my peers struggling, I thought, “I am not the only one going through this” or “It’s normal to face challenges.” Despite our struggles, we persevered, sometimes taking short naps when overwhelmed with fatigue, and waking each other up to prepare for presentations and work on our portfolios. Being in similar circumstances and facing challenges together, having such companions was a significant source of strength for me (Participant 5).

The deeper a student’s involvement in the entire process with a specific patient, and the closer the relationship with that patient, the more active the process of meaning formation became. However, as students grew closer to the patients, their sense of burden also increased.

One case that stands out vividly was of a patient who was airlifted. From the moment the helicopter touched down, I was hands-on with this case. The patient was in critical condition, and it was the first emergency case I had ever been witness. I had the unique opportunity to observe the entire process from the beginning to the end. I saw the patient being rushed for a transfusion, quickly taken to the operating room (where I had the chance to scrub in), and transferred to the discharge room, where I also saw the professors leave the patient. I also scrubbed in for a follow-up surgery. Such moments have left an indelible mark on my memory. While we usually observe cases for brief moments, this trauma patient was different. I spent a significant amount of time observing them, and to this day, the memory of that patient remains fresh in my mind (Participant 1).

The more closely I interacted with a patient, the more vividly I remember the experience. While observing a patient under the guidance of a professor is memorable, my most prominent memories are of directly communicating with the patient. Gaining experience on how to appear more competent, minimize harm, and choose the right tone in various situations was invaluable (Participant 2).

#### Inhibiting the negotiation

In the negotiation of meaning, participation and reification share a mutually complementary relationship. In the context of clinical clerkship, students’ learning was hindered when either participation or reification was overly emphasized. The negative impact of such overemphasis on participatory aspects was especially evident in students’ relationships with professors. This manifested as learning anxiety in the early stages of clerkship and feelings of alienation. In the initial stage of clerkship, where reification is often challenging to attain, students were passive in forming meaning. This could lead learners to perceive their learning as unstructured, introducing excessive cognitive and psychological burdens, and making meaning formation difficult. Furthermore, negative relational dynamics, such as professors’ indifference even beyond the initial stage, diminished opportunities for meaning formation by relegating students to positions of alienation rather than as peripheral participants.

During the early days of the clerkship, I felt such anxiety and concern that I found myself trying hard to avoid any reprimands from the professors. I often chose to stay in the background, remain quiet, and let things pass without posing questions. There was this nagging worry that if I did ask something, the professor might retort, “How can you not know such basic things?” This fear made me rather passive. (Participant 3).

In each early clinical clerkship session, I felt it would be helpful if there was a clear flow or direction for the content they wanted to convey to us. I’m not exactly sure how this can be achieved, but it’s something I believe could be improved (Participant 8).

Occasionally, during certain rotations or placements, we encountered professors who appeared indifferent to us. While it was their duty to guide us, it was clear that some weren’t genuinely interested in doing so. We felt somewhat neglected during such sessions, as if the guidance was merely a formality or an unpleasant task they had to get through. Those moments were particularly tough (Participant 2).

Situations that hindered the negotiation of meaning due to an excessive focus on reification were evident in the justice of the assessment system and the newly introduced e-portfolio. In contexts where meaningful mutual participation between learners and professors was challenging, issues of fairness and justice stemming from students’ lack of information led to a decline in motivation for ongoing learning and complicated the formation of meaning. Moreover, certain scaffolding structures in the new e-portfolio system proved to be barriers to effective meaning formation. Notably, the individualized e-portfolio system sometimes shifted too much focus to reification, risking the reproduction of decontextualized formats.

This situation was inevitably tricky. When we divided up the assignments, it might be overly burdensome for some, while others might feel less burdened. Besides, practical training is somewhat delicate because there were no clear assessment criteria. What professors expect from students also varies from one professor to another. As a result, one professor might say “You did very well” to everyone and give only half of the class a good grade. Another professor might say the same thing but give everyone a good grade. This kind of difference seems to be inevitable. So, feelings of subtle frustration and confusion seem to form part of clerkship (Participant 1).

Reflecting on it, I was indeed among those who took e-portfolio writing seriously. Even when peers would question, “Why are you writing so much detail?” I persisted because it felt natural and comfortable to me. There were various components to cover, such as the pre-diagnosis records. However, I found that neither the final reflection nor the weekly practice plans and reflections added much value for me. For the final reflection, I was tasked with documenting five aspects: beneficial experiences, an objective description of said experiences, my personal feelings about them, the impact they had on me, and my subsequent plans. This structure struck me as repetitive. After expressing my feelings about a reflection, I’d have to then elaborate on the influence of those feelings. And when outlining my future actions, I found myself revisiting and reiterating some of the same content. This redundancy became a point of contention. Many peers seemed to merely echo their previous points with slight variations. As such, I couldn’t help but think the approach lacked practicality (Participant 7).

## Discussion

This study delved into the detailed aspects of what and how medical students learn during their RBC. Using a sociocultural lens, we focused on the procedural aspects of RBC, which had previously been neglected. Our investigations allowed us to discern the educational meanings that emerge and to identify the educational environments that either foster or inhibit learning. Clinical clerkship, initiated by the act of “wearing a white coat” as an emblem of legitimate peripheral participation, crystallized into specific facets of becoming a doctor such as values, functionality, career decisions, sociality, and situating. These elements were subsequently harmonized and broadened into the overarching professional identity symbolized by the white coat.

The professional identity as a doctor is an expression of characteristics, values, and norms of the medical profession that have been internalized over time, culminating in thinking, acting, and feeling as a doctor [40]. The formation of this professional identity not only encompasses the competency-based educational goals aimed at producing primary healthcare professionals—a traditional objective of medical education—but also represents the ultimate direction pursued by medical education. This study identified five specific facets of professional identity based on learners’ experiences and further delineated them into individual and societal dimensions. From this, it became evident that learners absorb and evolve a wider range of elements during their RBC. These specific identities do not develop in isolation but evolve through ongoing, interconnected interactions.

**Fig. 1:**
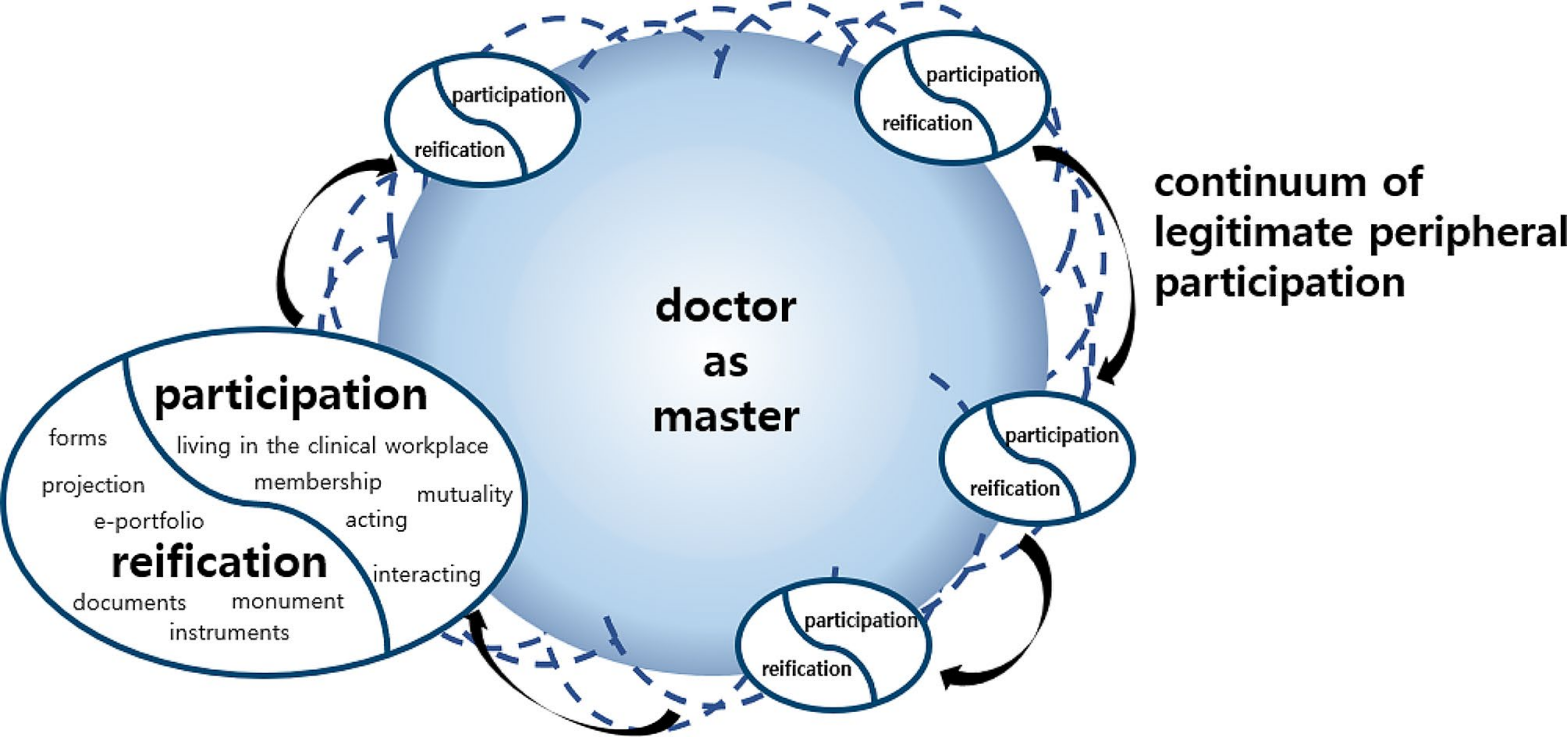
Legitimate peripheral participation and the process of learning in clinical clerkship. Note. Reorganization of Lave and Wenger’s legitimate peripheral participation [17] and Wenger’s negotiation of meaning model [42]

The process of forming a professional identity manifested as a continuum of legitimate peripheral participation, as shown in Fig. 1. Medical students, acting as peripheral participants in the healthcare workplace, received legitimacy as trainees. This allowed them to enhance their negotiation of meaning through both participation and reification. In particular, unlike Lave and Wenger’s situated learning theory, which view transition from the periphery to the core and identity change as learning [19], this study observed that learners consistently molded their identity even in scenarios where they persistently occupied a peripheral position. The cyclical nature of this periphery granted learners a unique “first experience” learning context, continuously fostering their learning enthusiasm. This played an integral role in the ongoing and dynamic negotiation of meaning in their professional environment. Such a phenomenon mirrors the unique aspect of traditional clinical clerkship, which adopts an RBC approach that consistently places students at the periphery. Through both participation and non-participation, students discerned the boundaries of a doctor’s identity. These results differ from a prior study that suggested that LIC could increase students’ sense of social connectedness due to the continuity of care, which may explain why students were more motivated to seek learning opportunities [41]. In other words, in addition to the fact that LIC is motivated by students’ relationship with other individuals, this study also found that the distinctive context of the first experience at an RBC was a further strong motivating factor for learning in clinical clerkship.

In the healthcare world, encounters with professors, peers, and patients have facilitated students’ RBC and aided in their negotiation of meaning. Praise from professors for positive behaviors, such as a learner’s excellence, and support in the face of negative behaviors, such as failures, have fostered trust in the relationship with the professors, thereby activating an autonomous negotiation of meaning. The genuine approach displayed by professors, not only in teaching but also in their own work, acted as a catalyst for the formation of learners’ identities. This observation aligns with the findings of previous studies on role modeling in medical education [43–46]. Given the nature of RBC, with its inherent time constraints and limited opportunities for deep relationships [47], there is a marked need to emphasize the authentic leadership of professors. Moreover, within a collaborative group culture, peers play a crucial role in shaping identity through a dual reciprocity relationship, serving both as monitors and supporters of one another’s reputation. This notion builds upon prior research, suggesting that peer support is an emotional facet of learning, as vital as the support from professors. Such support, in turn, has a positive influence on academic achievements [36–39]. Lastly, the more engaged learners are in their relationships with patients, and the less distance they perceive, the more vibrant their negotiation of meaning becomes. This finding is consistent with earlier research on patient-centered clerkship, particularly LIC. In other words, LIC’s focus on patient-centered care, which allows for continuity of care, high student satisfaction, and the potential for deeper learning in a consistent clinical context, also proved to be a critical context for learning in RBC [7, 48–49]. However, it is worth noting that while LIC can increase the contact time between professors, patients, and students, promoting the formation of meaningful relationships, it might also increase the emotional burden on learners, potentially obstructing their learning.

The non-learning environment that hinders the negotiation of meaning [21] arises when the dual practices of participation and reification lose harmony and tilt toward one or the other. Excessive participation, especially when forming new relationships with professors who are perceived as primary learning connections, can lead to initial anxiety and general feelings of alienation. When learners adjust to a new environment, they might experience anxiety due to insufficient interaction with others and the unfamiliar setting [50–51]. Enhanced interaction or the shared use of reified material between professors and students can mitigate anxiety. However, without timely educational intervention, the learners might exhibit a defensive silence stemming from fear, which can result in lost learning opportunities due to the absence of voice [52]. Especially in the RBC context, where students are ongoing peripheral participants, there is a distinct need for facilitating smooth organizational integration at the initial stages. Alienation in the professor–learner relationship can also pose difficulty engaging in negotiation of meaning, with professors’ indifference pushing learners from peripheral participants to excluded ones. Meanwhile, all learning is situated [19], and reification, when emerging after participation, can risk occurring out of its original context. To align it back, engaging in trust-based dialogues with key stakeholders in RBC becomes essential [53]. Potential solutions include setting up communication platforms for sharing implicit knowledge. Excessive reification manifested in evaluations and e-portfolio activities. Addressing this imbalance requires clear communication on assessment standards and enhancing participation, such as peer-sharing of e-portfolios and gathering professor feedback.

From a medical education perspective, the implications of this study are as follows: First, from an academic perspective, this research has concretized the professional identity formed by RBC learners into two dimensions: individual and societal. In this regard, we propose follow-up research to discern the learning contexts in which specific identities are strengthened. Particularly, the development of sociality occurs across both individual and societal dimensions of the acquisition process. This development can vary based on the considerations made during the interaction process and the nature of the interactive relationship [16]. Depending on whether an organizational culture leans more toward competitiveness or collaboration, learners’ identities are anticipated to exhibit discernable differences. Also, building on Wenger’s concepts of participation, peripherality, and exclusion [20], qualitative or comparative studies are recommended to investigate how specific identities evolve. It would be intriguing to study how professional identities are bolstered by experiences in extramural hospitals and clinics or through social roles assumed as a physician. Wenger posited that the core objective of learning is to expand into broader social communities [18]. This expansion of professional identity can be observed in students on the cusp of entering healthcare organizations, set against a backdrop of sociocultural dynamics, including social power relationships. Furthermore, given some evidence pointing toward the potential of deriving learning from learners’ unsuccessful experiences, future research may explore the phenomenon of learning from failure via qualitative case studies. Such research might align with transformative learning, thus contributing to identifying the necessary educational interventions that encourage reflective thought processes in learners.

Next, for those involved in clinical clerkship learning, we propose the following practical implications: First, it is essential to prioritize establishing an educational intervention that nurtures the formation of a meaningful relationship triad among professors, patients, and students. Beyond the initial benefits of the RBC experience, learners should have opportunities for direct and in-depth interactions with patients during their two-year clerkship, in line with LIC’s emphasis. Second, to help students foster a relationship with their professors as learning resources, we propose authentic leadership development for the professors and introducing reflective practice for curriculum committees. When the professors display authenticity in their professional engagements, they inspire learners to proactively shape their professional identity. Even if it is not feasible to extend the duration of tutor-learner interactions, the authenticity in forming relationships with learners is of crucial importance for promoting learning. The professors can utilize inter-professional education sessions to foster leadership. Inter-professional education training builds faculty members’ ability to effectively teach and role model actions to inter-professional students [54]. Meanwhile, curriculum committees can integrate pedagogical guidelines such as the one-minute preceptor [55] into the clerkship curriculum. Students can utilize the allocated time to engage in reflection which in turn promotes learning through participation [56–57]. Finally, it is necessary to build trust by addressing the shortfall in explicit knowledge and enhancing participation in assessments and e-portfolios to bridge communication gaps. Curriculum committees may consider utilizing a group method of integrating reflective practice [58]. Particularly, there is a need to prepare physical platforms for open sharing of reification among new and experienced students, as well as between learners and professors, or educational interventions, such as communication arenas.

This study presents the analysis results of basic qualitative study of sixth-year medical students in South Korea. While this research design offers the advantage of enabling an in-depth exploration of specific learners’ experiences, its findings cannot be directly generalized to all medical students, and neither is generalizability the objective of qualitative research. Nevertheless, based on the universal findings derived from this study, we were able to empirically approach the essence of RBC. The findings of this study are expected to contribute to future improvements in clinical clerkship education.

## Conclusions

This study attempted to view the educational process of a clinical clerkship, which has not been focused on so far, from the perspective of learners. Since the birth of the clerkship is based on a form of apprenticeships, Lave and Wenger’s situated learning theory and Wenger’s social theory in learning, which explain learning in apprenticeships, were used as a lens for interpretation. Consequently, it was possible to understand what medical school students learn through RBC, how they learn, and contexts that promote or hinder a negotiation of meaning during the learning process. RBC’s unique process of professional identity formation and five sub-identities, namely values, functionality, career decision, sociality, and situating, formed as a result of it were revealed. Students were constantly situated on the periphery, learning through a combination of participation and reification. In this process, first experiences and relationships with others acted as facilitators of learning in RBC. In addition, excessive participation or reification behavior was found to cause non-learning situations in RBC such as alienation, injustice in assessment, and technical writing of e-portfolio. Therefore, this study helps to contribute to the gradual improvement of traditional RBC and the provision of learner-centered educational clerkship programs rather than a transition to LIC or a rapid change despite cost-effectiveness and feature issues.

## Data Availability

The datasets of this article are available from the corresponding author upon reasonable request.

## Abbreviations

LIC: Longitude Integrated Clerkship
RBC: Rotation-Based Clerkship
CoP: Communities of Practice
IRB: Institutional Review Board
